# Clinical form of babesiosis caused by *Babesia canis* in Polish foxes (*Vulpes vulpes*)

**DOI:** 10.1007/s11259-025-11028-9

**Published:** 2026-01-08

**Authors:** Łukasz Adaszek, Jagoda Ciszewska-Ceran, Maria Pisarek, Banu Dokuzeylül, Mehmet Erman Or, Maciej Skrzypczak, Marcin Kalinowski, Beata Horecka, Andrzej Jakubczak, Stanisław Winiarczyk

**Affiliations:** 1 Department of Epizootiology and Infectious Diseases, Faculty of Veterinary Medicine, University of Life Sciences in Lublin, 30 Głęboka St. 20-612, Lublin, Poland; 2 Sub-Department of Diagnostics and Veterinary Dermatology, Faculty of Veterinary Medicine, University of Life Sciences in Lublin, 30 Głęboka St, 20-612 Lublin, Poland; 3 Department of Internal Medicine, Veterinary Faculty, Istanbul University- Cerrahpasa, 34320 Avcilar Campus, Avcilar, Istanbul, Turkey; 4 Chair and Department of Gynaecology, Medical University of Lublin, 8 Jaczewskiego St, 20-954 Lublin, Poland; 5 Institute of Biological Basis of Animal Production, University of Life Sciences in Lublin, 20-950 Lublin, Poland

**Keywords:** Babesia canis, Foxes, Ticks, Vector-borne diseases

## Abstract

The red fox (*Vulpes vulpes*) is known to be a reservoir host of various vector-borne protozoan parasites. However, the impact of infections caused by *Babesia canis* on the health status of the red fox remains unknown, and research on this topic conducted on fox populations in Poland and worldwide has been fragmentary. It is known that these animals can become infected with *Babesia canis*, but it is unclear whether a clinical form of the disease can develop in them. This study aimed to present for the first time the cases of clinical babesiosis in foxes in Poland. The observations covered four foxes aged 3–6 years with apathy, anemia, brown color of urine and thrombocytopenia. The PCR and sequencing results confirmed that all the animals had been infected with *Babesia canis* protozoa. The diagnosis of clinical cases of babesiosis due to *B. canis* in foxes from this region of Europe suggests a contribution of red foxes to the establishment of this animal species as a new reservoir of *B. canis*. These animals may play a role as a host for *B. canis*, and previously unexposed red fox populations may be more prone to infection in areas colonized by *D. reticulatus*.

## Background

*Babesia canis* is a common and clinically significant hematozoan parasite of the genus *Babesia*, transmitted by ticks (Mehlhorn et al. 1980; Adaszek et al. 2009). *Babesia* spp. are classified in the order of Piroplasmida within the phylum Apicomplexa. In early studies, researchers identified two morphologically distinct forms of the erythrocytic stage in the canine host. The larger form, measuring approximately 3–5 μm, was named *B. canis*, while the smaller form (1–3 μm), was named *B. gibsoni* (Adaszek et al. 2010). Based on cross-immunity, serological testing, vector specificity and molecular phylogeny, *Babesia canis* was reclassified into three separate species (*B. canis*, *B. rossi* and *B. vogeli*) (Carret et al. 1999; Costa-Júnior et al. 2009; Zygner et al. 2023).

The red fox (*Vulpes vulpes*) is known to be a reservoir host of various vector-borne protozoan parasites. Among the different *Babesia* species detected in foxes, the most frequently identified parasites are *B. vulpes* (the frequency of detection ranges from 69.2% in Portugal, 64.1% in the Czech Republic, 20% in Hungary, 13.6% in Italy, and 0.8% in Bosnia and Herzegovina) (Cardoso et al. 2013; Hodžić et al. 2015; Farkas et al. 2015; Ebani et al. 2022; Lesiczka et al. 2023)d *vogeli* (3% in France) (Medkour et al. 2020).

The impact of infections caused by *B. canis* on the health status is unknown (Cardoso et al. 2013; Lesiczka et al. 2023), and research on this topic carried out on the fox population in Poland has been fragmentary. It is known that these animals can become infected with the parasites concerned, although it is not known whether a clinical form of the disease can develop in them (Dwużnik et al. 2020; Mierzejewska et al. 2021).

The current study aimed to present four cases of clinical *Babesia canis* infections in foxes in Poland.

## Case presentation

The observations were conducted between June and November 2024 and included four red foxes (three females and one male, Nos 1–4) aged between 3 and 6 years, which were presented to the Clinic of the Faculty of Veterinary Medicine at the University of Life Sciences in Lublin, Poland. Three animals (two females and a male) originated from private zoological gardens in eastern Poland, whereas one animal (a female) was kept as a companion animal (it was kept outdoors, the household was located near the forest, and the fox had permanent contact with ticks). The keepers of all the foxes had observed ticks on their body integuments. In the case of two animals, the acarids were brought to the clinic, where they were identified and determined to be *Dermacentor* based on the standard morphological keys (Nosek and Sixl 1972; Földvári et al. 2016). In all animals, a clinical examination as well as hematological and biochemical blood examinations were performed. The study was conducted in accordance with the Directive of the European Parliament on the Protection of Animals Used for Scientific Purposes (Directive 2010/63/EU), and all owners of the foxes agreed to their inclusion in the study. Blood sampling was a part of the clinical procedure, and no local ethics committee approval was required. Blood was also collected from all animals for molecular testing for babesiosis, using the forward primer BAB GF2 (5’-GTC TTG TAA TTG GAA TGA TGG-3’), and the reverse primer BAB GR2 (5’-CCA AAG ACT TTG ATT TCT CTC-3’), which amplify a 559-bp region of the *18 S rRNA* gene of *Babesia* spp. (Bonnet et al. 2007). DNA was extracted from EDTA-anticoagulated whole blood using the Blood Mini DNA isolation kit (A&A Biotechnology, Gdansk, Poland) according to the manufacturer’s instructions.

The PCR mixture (50 µL) for *Babesia* contained 100 µM of each dNTP, 1.6 mM MgCl₂, 0.25 µM of each primer, 2.5 U of Taq DNA recombinant polymerase (Thermo Fisher Scientific U.S.), and 5 µL of DNA template. PCR amplification was performed in a programmable thermal cycler (Biometra, Göttingen, Germany) under the following conditions: initial denaturation at 92 °C for 2 min; 50 cycles of denaturation at 92 °C for 60 s, annealing at 52 °C for 60 s, and extension at 72 °C for 90 s; followed by a final extension at 72 °C for 5 min. PCR products were analyzed by electrophoresis in a 1% agarose gel stained with ethidium bromide, alongside a 100-bp DNA ladder (Gibco/BRL, Gaithersburg, MD, USA).

The PCR products were then purified using QIAquick spin columns (Qiagen), eluted in 50 µL of Tris 10 mM, pH 7.6, and sequenced in the Research Institute, Polish Academy of Sciences, Warsaw, Poland. DNA sequences were assembled and edited using SeqMan (DNAstar, Lasergene, Madison, USA). BLAST analysis was performed for all sequences using the online tool Nucleotide BLAST (https://blast.ncbi.nlm.nih.gov/Blast.cgi). Each sequence was compared individually to database resources, and representative results showing top three matches are presented in the Table 1.

**Table 1:** Results of sequence alignment to reference sequences using BLASTn

Query	Scientific name	Identity	Query cover	E-value	Accession
Fox 1–4	*Babesia canis*	100%	100%	0.0	MK070118
	*Babesia canis*	100%	100%	0.0	KY447296
	*Babesia canis canis*	100%	100%	0.0	MK571830

All blood samples were also analyzed in a BIONOTE Vcheck M10 analyzer (VetExpert, Poland), for *Leishmania* spp., *Leptospira* spp., *Babesia* spp., *Mycoplasma haemocanis*,* Hepatozoon* spp., *Ehrlichia canis*,* Anaplasma* spp. *and Bartonella* spp. in a real-time PCR (Canine Vector 8 Panel, Canine Anemia 8 Panel). The results of these analyses were positive only for *Babesia spp*.

**Fig. 1:**
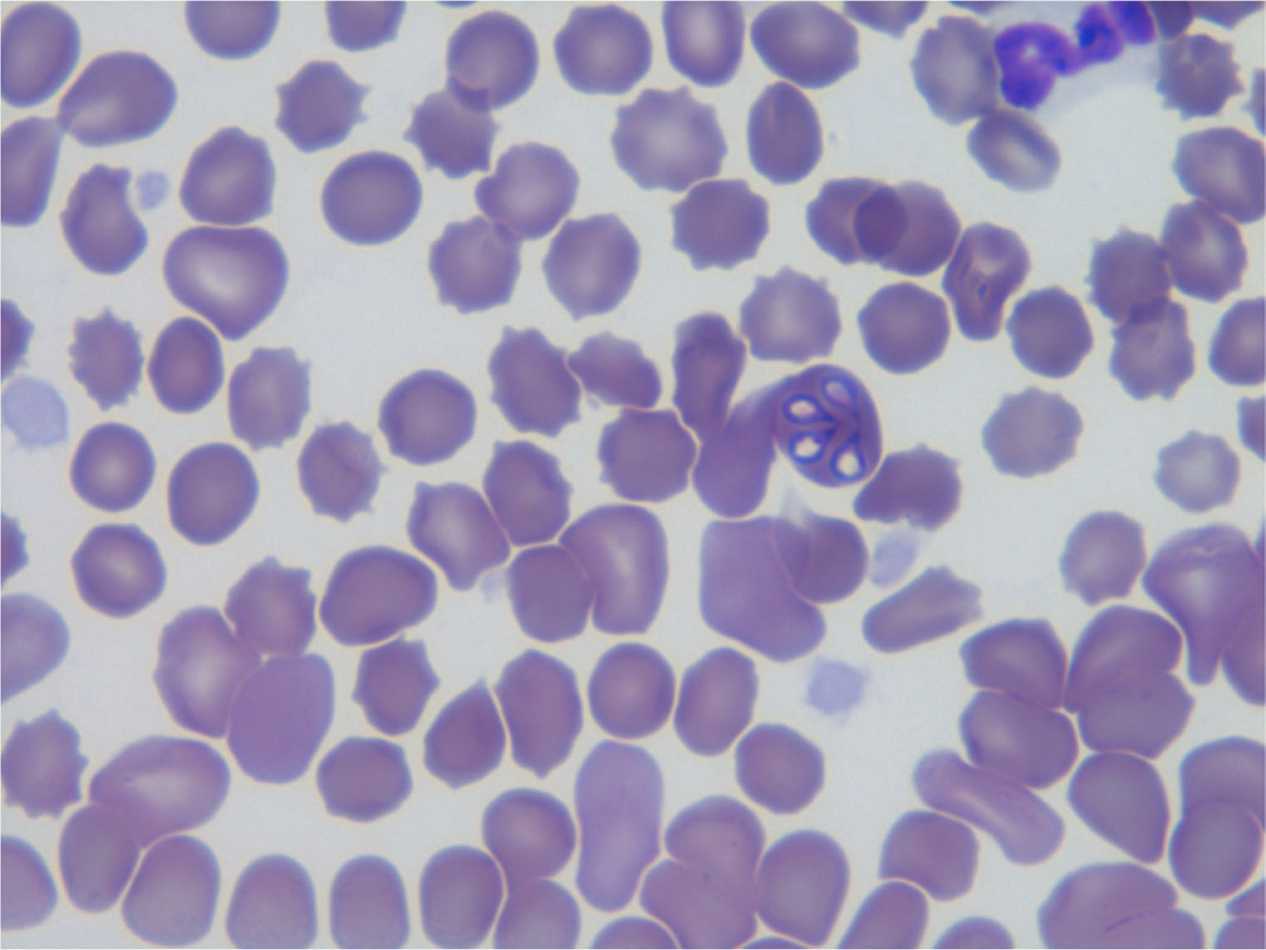
Presence of *Babesia* merozoites in the red blood cells of fox No. 3

All foxes were apathetic and anemic. Animals refused to drink water or eat. Mucous membranes (oral cavity and conjunctiva) were pale. In three of them, a brown color of the urine was observed. No pain was observed on abdominal palpation. In two animals (No. 2 and No. 3), an increase in body temperature was noted, while tachypnoe was recorded in all of them (Table 2). A hematological examination revealed anemia and thrombocytopenia in all of the animals. The hematology and biochemistry results concerning the reference values (Benn et al. 1986) are shown in Tables 3 and 4. Microscopic analysis of the blood smears revealed the presence of *Babesia* spp. merozoites in the red blood cells of two foxes (No. 3 and No. 4) (Fig. 1).

**Table 2:** Body temperature, heart rate and respiratory rate in foxes infected with *B. canis* in relation to reference ranges (Kreeger et al. 1989)

No	Body temperature (°C)	Heart rate (beats/min)	Respiratory rate (respirations per min)
001	38.8	134	24
002	41.1	142	32
003	40.7	141	35
004	39.6	135	26
Range	38.7–40.5	130–150	16–20

**Table 3:** Results of the hematological examinations of foxes

No	WBC^a^ (x 10^9^)	RBC^b^ (x 10^12^)	Ht^c^ (%)	MCV^d^	MCH^e^	MCHC^f^	PLT^g^ (x 10^9^)
001	6.4	6.22	38.2	67.1	24.4	36.4	123
002	5.7	7.04	33.1	69.4	25.9	37.4	199
003	4.8	4.20	29.8	73.5	24.8	33.8	241
004	4.4	5.34	31.4	70.8	25.2	35.7	121
Range	3.5–15	9.1–12.5	39–57	62–78	21–28	30–38	266–855

^*a*^*WBC* White Blood Cells, ^*b*^*RBC* Red Blood Cells, ^*c*^*Ht* Hematocrit, ^*d*^*MCV* Mean Corpuscular Volume, ^*e*^*MCH* Mean Corpuscular Hemoglobin, ^*f*^*MCHC* Mean Corpuscular Hemoglobin Concentration, ^*g*^*PLT* Platelets

**Table 4:** Results of the serum biochemical examination of foxes

No	ALT^a^ (IU)	AST^b^ (IU)	BIL T^c^ (mg/dL)	ALP^d^ (IU)	UREA (mg/dL)	CREATININE (mg/dL)
001	84	41	0.36	121	83.3	0.72
002	30	27	0.51	96	79.8	1.34
003	45	69	0.32	122	84.2	1.12
004	72	55	0.70	77	91.6	1.77
Range	5–157	19–76	0–0.9.9	28–129	20–77	0.5–1.8

^*a*^*ALT* Alanine Aminotransferase, ^*b*^*AST* Aspartate Aminotransferase, ^*c*^*BIL T* Total Bilirubin, ^*d*^*ALP* Alkaline Phosphatase

*Babesia* DNA was found in blood samples from all four ill foxes. Based on similarities between sequences of *18 S rRNA* gene fragments, all four samples were classified as the *B. canis*. They showed a 100% homology with *B. canis* sequences deposited in GenBank database (Table 1).

The animals were injected subcutaneously with an imidocarb dipropionate solution (Imizol^®^ Schering Plough Animal Health) in a single dose of 3 mg/kg. Twenty-four hours after the application of the drug, an improvement in the clinical status was observed in all animals. A control PCR test carried out two weeks after the previous test (using the same procedure) did not reveal genetic material of *Babesia* in the animal’s blood.

## Discussion and conclusions

The present study has demonstrated clinical cases of babesiosis caused by *B. canis* parasite in red foxes from eastern Poland. Parasitic infections of this protozoan species are not common in representatives of these animals. The primary species of the *Babesia* protozoan, found in the European fox population, is *B. vulpes*, whereas infections caused by *B. canis* are noted rather sporadically. The prevalence of these infections in the Serbian fox population was only 0.8% (Juwaid et al. 2019). In Poland, these values were 2.4% (Mierzejewska et al. 2021). In Portugal, the detection frequency of *B. canis* was only 1.1% (Cardoso et al. 2013). In Germany (Liesner et al. 2016), the United Kingdom (Bartley et al. 2016) and Italy (Zanet et al. 2014), no presence of *B. canis* was noted in any of the foxes studied for piroplasm infections. This indicates that *B. canis* infections in the red fox population are relatively uncommon. It should be noted that in all the studies cited above, protozoa were detected in tissues collected from necropsied foxes; therefore, it was not possible to determine whether these infections had led to the development of clinical symptoms in the animals. Nevertheless, the diagnosis of clinical cases of babesiosis in foxes in Poland suggests a higher susceptibility of the red fox population to a novel introduced pathogen in areas colonized by *Dermacentor reticulatus*.

The described cases of babesiosis caused by *B. canis* in foxes from eastern regions of Poland correspond to the occurrence of the main vector of these protozoa, i.e. the tick *D. reticulatus*, in this area. This tick species was found on the body of two of the four foxes exhibiting clinical signs of babesiosis, which may indirectly indicate their involvement in the disease transmission.

All of the observed foxes exhibited clinical signs characteristic of canine babesiosis, and hematological examinations revealed typical abnormalities associated with the disease, including thrombocytopenia and anemia (Adaszek et al. 2009). Significant reductions in red blood cell count and hemoglobin concentration are caused by mechanical damage to erythrocytes during parasite egress, intravascular hemolysis, and immune- or non-immune-mediated erythrocyte destruction (Zygner et al. 2007). The mechanism of thrombocytopenia has not been fully elucidated. One possible explanation is platelet sequestration in the spleen, immune-mediated platelet destruction, or the development of disseminated intravascular coagulopathy (DIC) (Schetters and Eiling 1999). Increased urea levels during babesiosis may indicate hemolysis, severe anemia, or renal failure (Lobetti and Jacobson 2001; Zygner and Gójska-Zygner 2014).

Similar hematological disorders and clinical symptoms to those observed in foxes in our study have also been reported in dogs with anaplasmosis, ehrlichiosis, bartonellosis, or hepatozoonosis (Adaszek et al. 2009, 2024; Mazurek et al. 2019; Teodorowski et al. 2021; Dokuzeylül et al. 2024). For this reason, these diseases, as well as hemotropic mycoplasmosis, leishmaniasis, and leptospirosis, were considered in the differential diagnosis; however, negative results were obtained for all of them.

Sequencing of the genetic material derived from the blood of infected foxes showed 100% homology with multiple reference sequences of *B. canis.* Among these was sequence obtained from dog in Poland (EU62279). Identical *18 S rRNA* sequences of *B. canis* were also detected in dogs with acute babesiosis, and in *Dermacentor reticulatus* ticks in our previous studies in Poland (Adaszek and Winiarczyk 2008; Adaszek et al. 2009, 2024; Ghodrati et al. 2025).

The current study suggests that red foxes may serve as a host for *B. canis*, and naïve populations of this species may be more susceptible to infection in new areas colonized by *D. reticulatus* (Zygner et al. 2009; Mierzejewska et al. 2021). The description of the presented cases further indicates that *B. canis* infection should be considered in the differential diagnosis of red foxes in Poland and Europe, especially in individuals with thrombocytopenia associated with *D. reticulatus* parasitism. Previous studies have primarily focused on the epidemiology of *D. reticulatus* in red foxes as a vector for *B. vulpes* (Mierzejewska et al. 2021). The current findings indicate a potentially increasing role of *B. canis* in fox infections; however, further studies are required to determine whether red foxes can serve not only as incidental hosts but also as reservoirs capable of maintaining this haemoprotozoan parasite. A better understanding of the epidemiology of ectoparasites, such as *D. reticulatus*, their role in pathogen transmission, and their impact on the health of wild animals is crucial for effective management of fox populations and reducing the risk of babesiosis spreading in this animal species.

## Data Availability

All data generated or analysed during this study are included in this published article.
